# Circular RNA F-circEA-2a derived from *EML4-ALK* fusion gene promotes cell migration and invasion in non-small cell lung cancer

**DOI:** 10.1186/s12943-018-0887-9

**Published:** 2018-09-20

**Authors:** Shuangyan Tan, Dan Sun, Wenchen Pu, Qiheng Gou, Chenglin Guo, Youling Gong, Jiao Li, Yu-Quan Wei, Lunxu Liu, Yun Zhao, Yong Peng

**Affiliations:** Key Laboratory of Bio-Resource and Eco-Environment of Ministry of Education, College of Life Sciences; Department of Thoracic Surgery, State Key Laboratory of Biotherapy, West China Hospital, Sichuan University, Chengdu, 610041 China

**Keywords:** Non-small cell lung cancer, EML4-ALK, Circular RNA, Cell migration/invasion

## Abstract

Oncogenic fusion gene Echinoderm Microtubule-associated protein-Like 4-Anaplastic Lymphoma Kinase (*EML4-ALK*) contributes to tumorigenesis of a subset of non-small cell lung cancer (NSCLC). Recently, we demonstrated that F-circEA-4a, a tumor-promoting circular RNA (circRNA) generated from the back-splicing of *EML4-ALK* variant 3b (v3b), is a novel liquid biopsy biomarker for NSCLC. However, circRNAs produced from *EML4-ALK* gene and their roles in NSCLC are not well-characterized. Here, we identify another *EML4-ALK*-v3b-derived circRNA, F-circEA-2a, harboring “AA” (rather than “AAAA” in F-circEA-4a) motif at the junction site. F-circEA-2a mainly locates in the cytoplasm and promotes cell migration and invasion, but has little effect on cell proliferation. Moreover, F-circEA-2a exists in tumor, but not in the plasma of NSCLC patients with *EML4-ALK* fusion gene, further supporting the significant diagnostic value of F-circEA-4a for *EML4-ALK*-positive NSCLC. This work finds a novel oncogenic circRNA generated from *EML4-ALK* fusion gene, highlighting the pivotal role of circRNA in *EML4-ALK*-positive NSCLC development.

**Electronic supplementary material:**

The online version of this article (10.1186/s12943-018-0887-9) contains supplementary material, which is available to authorized users.

## Main text

Lung cancer is the leading cause of cancer death worldwide, in which non-small cell lung cancer (NSCLC) is a main subgroup accounting for approximately 85% of all lung cancer cases [1]. NSCLC patients are often diagnosed at advanced stage and their 5-year survival rate is extremely low [2]. Thus, investigation of NSCLC-associated process is urgent for NSCLC diagnosis and treatment.

A subset of NSCLC harbor fusion gene which encodes fusion protein to exert oncogenic phenotype. For example, Echinoderm Microtubule-associated protein-Like 4-Anaplastic Lymphoma Kinase (*EML4-ALK*) fusion gene is present in 4–5% of NSCLC cases and generates *EML4-ALK* fusion protein to activate ALK-associated oncogenic signaling and promote NSCLC progression [3–5]. However, their underlying mechanism in NSCLC remains obscure.

Circular RNAs (circRNAs) are a special subtype of non-coding RNAs with circular covalently-bonded structure, which endows them higher tolerance to exonucleases. Due to their conservation, abundance and specificity, circRNAs participate in diverse physiological and pathological processes, including tumorigeneisis [6]. Increasing evidence demonstrate that circRNAs derived from the back-splicing of fusion gene are alternative entities involved in cancer development besides fusion proteins. For instance, the circRNA generated by *MLL/AF9* fusion gene (f-circM9) in leukemia shows pro-proliferative and pro-oncogenic activities [7]. Additionally, we recently demonstrated that fusion gene *EML4-ALK* variant 3b (v3b) produces an oncogenic circRNA (F-circEA-4a) with “AAAA” motif at the junction site. Importantly, F-circEA-4a could be detected in the plasma of *EML4-ALK*-positive NSCLC patients, acting as a potential liquid biopsy biomarker [8]. However, circRNA-producing potential of *EML4-ALK* gene and the role of these circRNAs in NSCLC are not fully understood.

Here, we identify another circRNA F-circEA-2a produced from *EML4-ALK*-v3b with “AA” motif at the junction site. Moreover, F-circEA-2a has little effect on cell proliferation, but promotes cell migration and invasion in NSCLC cells, highlighting the critical role of circRNAs in *EML4-ALK*-positive NSCLC.

## Results and discussion

### Identification of F-circEA-2a in NSCLC

**Fig. 1:**
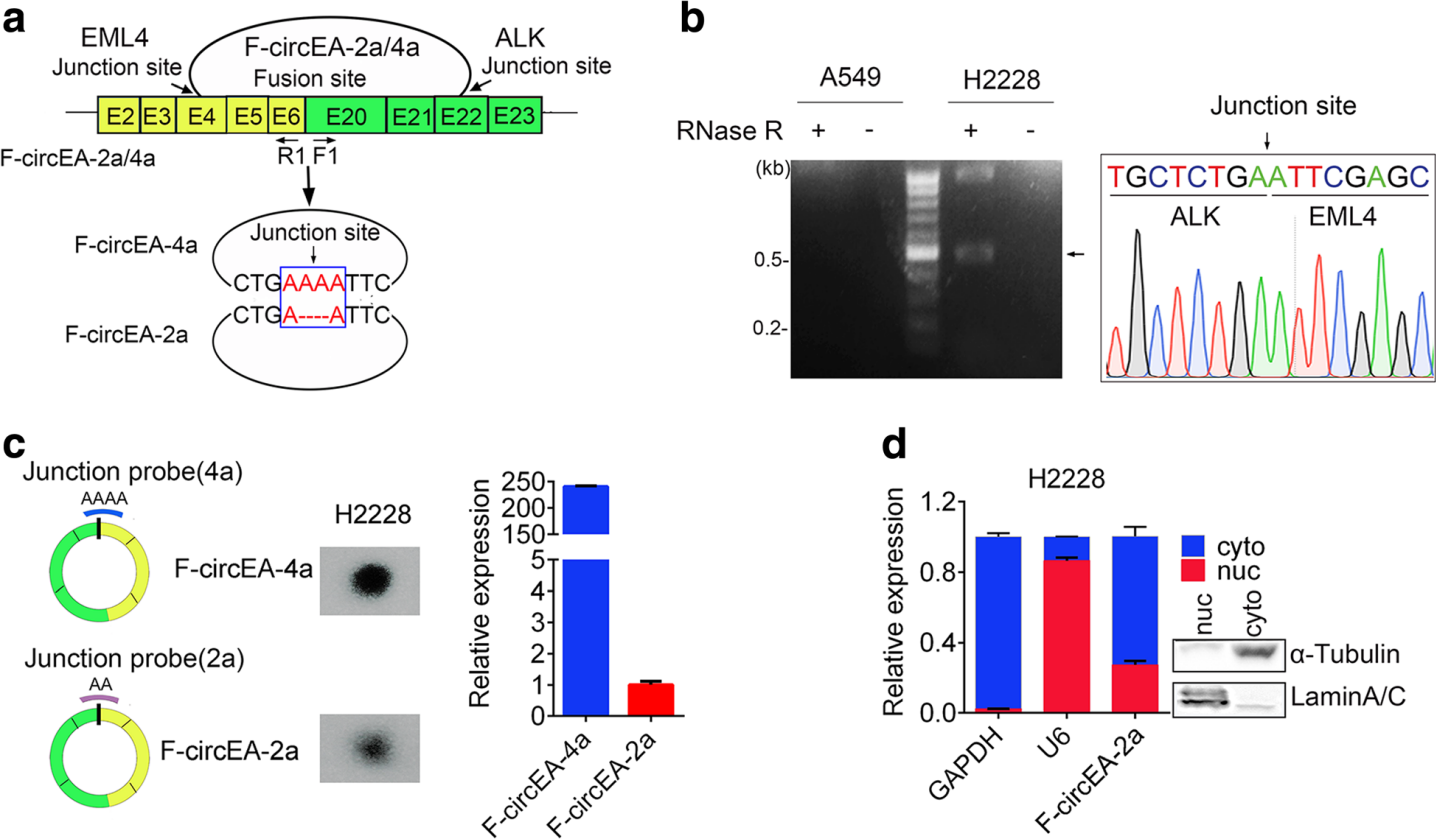
Identification of F-circEA-2a in NSCLC. **a** Schematic representation of F-circEA-2a/4a generated from *EML4-ALK* gene. The divergent primers (F1/R1) were used to detect F-circEA-2a/4a. **b** RT-PCR and Sanger sequencing of F-circEA-2a in H2228 cells. The arrow indicates the junction site of F-circEA-2a. **c** Measurement of F-circEA-2a/4a in H2228 cells by dot blot hybridization (left) and qPCR (right, normalized to GAPDH mRNA). **d** qPCR analysis of F-circEA-2a after nucleus/cytoplasm fractionation of H2228 cells. GAPDH mRNA and U6 RNA were used to indicate the cytoplasmic and nuclear RNA, respectively. Western blotting confirmed good nucleus/cytoplasm fractionation. Data are shown as the mean ± SD. See Additional file 1: Table S1 for the information of the primers and oligonucleotides in this figure. The experimental protocols are described in the Additional file 2

We recently identified the existence of *EML4-ALK*-derived circRNA F-circEA-4a. To evaluate whether *EML4-ALK* fusion gene could produce other circRNAs, we deeply Sanger-sequenced the reverse transcription PCR (RT-PCR) products using divergent F1/R1 primers (Fig. 1a) from H2228 (harboring EML4-ALK-v3b) and A549 cells (without the fusion gene, negative control). Besides F-circEA-4a, we found the presence of another circRNA named as F-circEA-2a with “AA” motif at the junction site in the RNA sample from H2228 cells, which was treated with RNase R to remove linear RNAs (Fig. 1b). F-circEA-2a, about 0.55 kb in length, is the back-splicing product between 5′-head of *EML4* exon4 and 3′-tail of *ALK* exon22 (Fig. 1a). Dot blot hybridization using ^32^P-labeled probes across the respective junction sites indicated that the enrichment of F-circEA-2a in H2228 cells was less than that of F-circEA-4a (Fig. 1c, left), which was further confirmed by quantitative PCR (qPCR) (Fig. 1c, right). Subcellular fractionation and qPCR assays showed that F-circEA-2a was mainly located in the cytoplasm (Fig. 1d). These data demonstrated the presence of another circRNA F-circEA-2a produced from *EML4-ALK* fusion gene.

### F-circEA-2a promotes cell migration and invasion in NSCLC cells

**Fig. 2:**
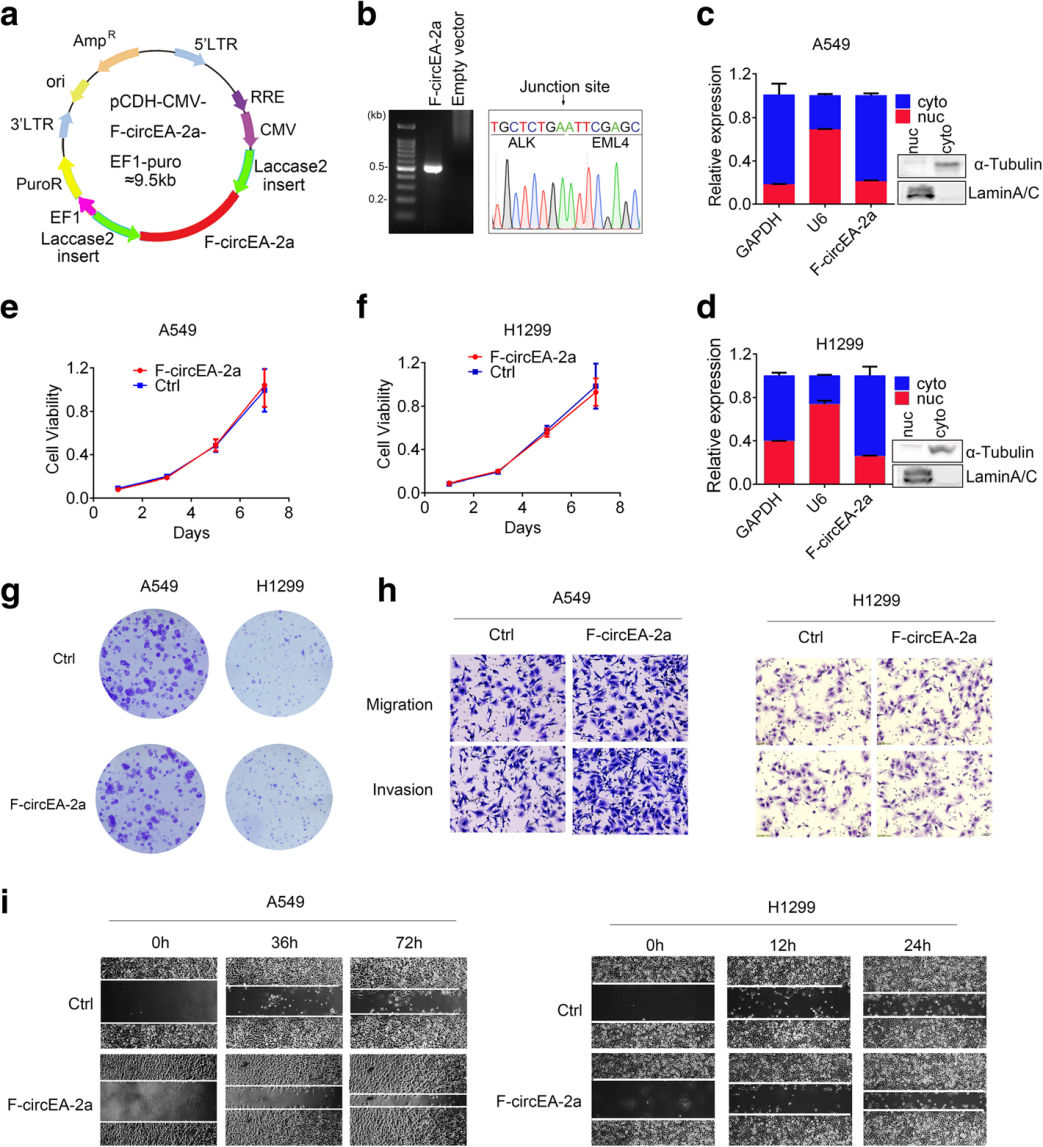
F-circEA-2a promotes cell migration and invasion in NSCLC cells. **a** Schematic representation of F-circEA-2a-expressing plasmid with the flanking sequence of laccase 2 to facilitate RNA circularization. **b** Agarose gel electrophoresis and Sanger sequencing of RT-PCR products from H1299 cells transfected with F-circEA-2a-expressing plasmid and empty vector. **c.d** Nucleus/cytoplasm fractionation and qPCR analysis of F-circEA-2a in A549 (**c**) and H1299 (**d**) cells. Western blotting against laminA/C and tubulin showed efficient nucleus/cytoplasm fractionation. Data are shown as the mean ± SD. **e.f.g** MTT (**e.f**) and colony formation assays (**g**) in A549 and H1299 cells transfected with F-circEA-2a-expressing plasmid or empty vector (Ctrl). **h.i** Representative images of Transwell (**h**) and wound-healing assays (**i**) in A549 and H1299 cells transfected with F-circEA-2a-expressing plasmid or empty vector (Ctrl). See Additional file 1:Table S1 for the information of the primers and oligonucleotides in **a** and **b**. The experimental protocols are described in the Additional file 2

To investigate cellular function of F-circEA-2a, we constructed F-circEA-2a-expressing plasmid, in which F-circEA-2a sequences (red arrows) and the flanking sequences (green arrows, favorable for circRNA formation) were cloned into pCDH-CMV-MCS-EF1-puro vector (Fig. 2a). Sanger sequencing of RT-PCR products from the cells transfected with F-circEA-2a-expressing plasmid indicated that F-circEA-2a was successfully expressed and correctly back-spliced (Fig. 2b). The ectopically expressed F-circEA-2a was predominantly located in the cytoplasm of both H1299 and A549 cells (Fig. 2c and d), same as the endogenous one in H2228 cells.

Both MTT and colony formation assays suggested that ectopically expressed F-circEA-2a had no significant effect on cell proliferation in A549 and H1299 cells (Fig. 2e, f and g). However, F-circEA-2a enhanced cell migration and invasion in both cells by Transwell assays and wound-healing experiments (Fig. 2h and i). Because both F-circEA-4a and F-circEA-2a can promote cell migration and their difference is that F-circEA-4a has extra AA dinucleotides at the junction site, so they may exert the cellular function through the same mechanism. Emerging evidence shows that circRNAs play an important role under physiological or pathological conditions. For example, circMTO1, down-regulated circular RNA in hepatocellular carcinoma (HCC), suppresses HCC progression by acting as the sponge of oncogenic miR-9 to promote p21 expression [9]. These data reveal a novel circRNA F-circEA-2a enhances cell migration and invasion without any influence on cell proliferation, enlarging the understanding of circRNA as oncogenic molecule participating tumor development.

### Detection of F-circEA-2a in NSCLC patient

**Fig. 3:**
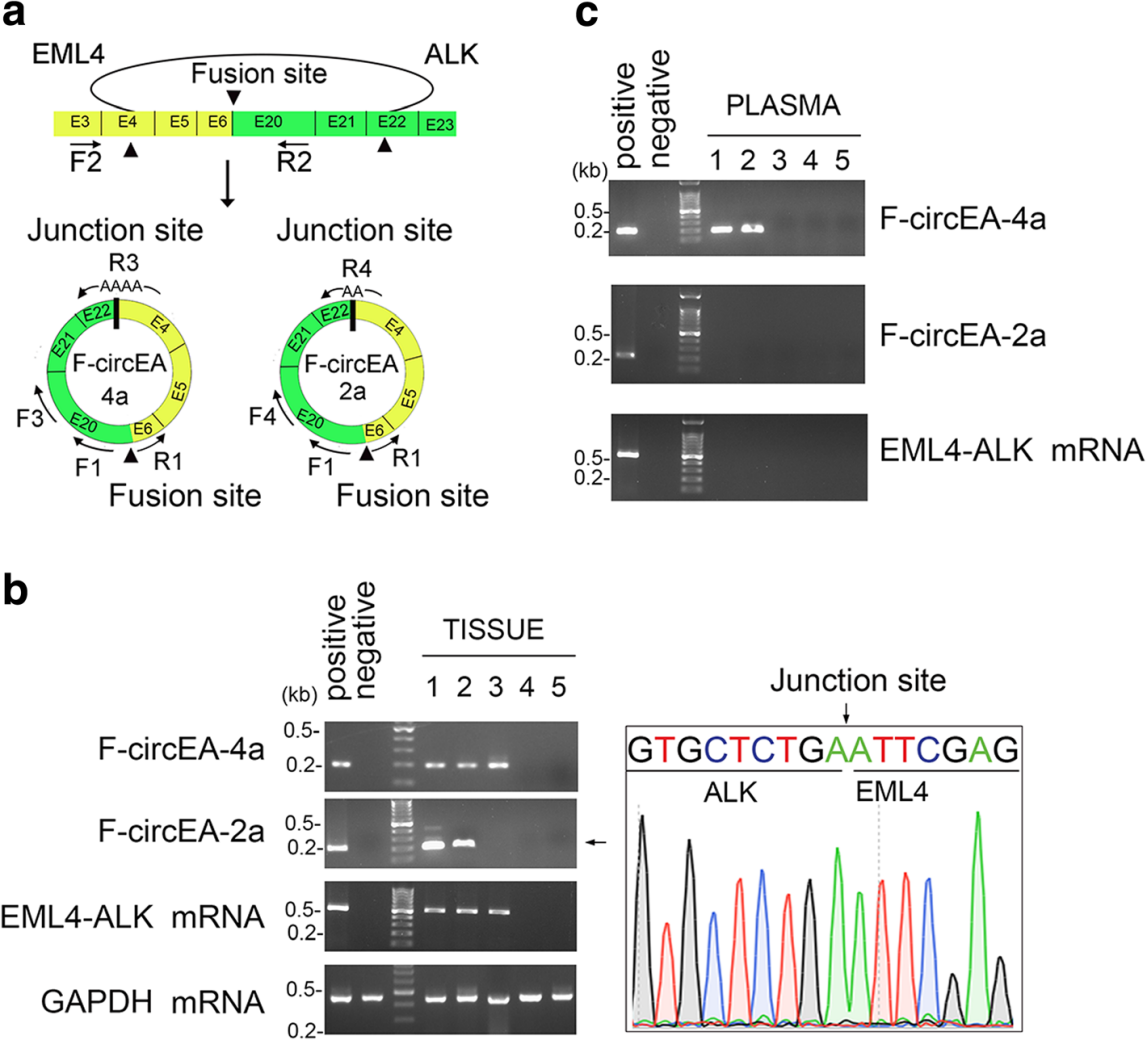
Identification of F-circEA-2a in NSCLC patients’ samples. **a** Primers used to detect *EML4-ALK* mRNA and F-circEA-2a/4a. The convergent primers (F2/R2) were used to detect *EML4-ALK* mRNA, the divergent primers F3/R3 and F4/R4 were used to detect F-circEA-4a/2a, respectively. **b.c** Agarose gel electrophoresis and Sanger sequencing of RT-PCR products from tumor tissues (**b**) or plasma (**c**) of NSCLC patients with (patients 1–3) or without (patients 4–5) *EML4-ALK* variant 3b translocation. See Additional file 1:Table S1 for the information of the primers and oligonucleotides in this figure. The experimental protocols are described in the Additional file 2

Compared to linear RNAs, the covalently-bonded circRNAs are relatively resistance to RNase R digestion. Some circRNAs (such as circ-N4BP2L2, circ-GSE1) originated from cancer tissues may enter the circulating system and stably be present in intercellular fluid [10]. Our recent work indicates that F-circEA-4a is a potential liquid biopsy biomarker for *EML4-ALK*-positive NSCLC patients [8]. To investigate the clinical significance of F-circEA-2a, we measured F-circEA-2a levels in tumor tissues and plasma of three NSCLC patients with *EML4-ALK-*v3b translocation and two NSCLC patients without such fusion gene. The convergent primers (F2/R2) were also used to detect *EML4-ALK* fusion mRNA. To improve specificity, we designed nested divergent primers (Fig. 3a, first round PCR primers: F1/R1; nested PCR primers for F-circEA-4a: F3/R3, nested PCR primers for F-circEA-2a: F4/R4). The R3 and R4 primers were designed to cross the junction sites of F-circEA-4a and F-circEA-2a, respectively, facilitating specific detection of these circRNAs (Fig. 3a). RT-PCR and Sanger sequencing data demonstrated that F-circEA-4a, F-circEA-2a and *EML4-ALK* mRNA were all detected in the tumor tissues of NSCLC patients with *EML4-ALK*-v3b translocation, whereas they were absent in the patients without such fusion gene (Fig. 3b), indicating the specific existence of F-circEA-4a and F-circEA-2a in the *EML4-ALK*-positive NSCLC tumors. However, in contrast to F-circEA-4a, we failed to detect F-circEA-2a in the plasma of *EML4-ALK*-positive NSCLC patients (Fig. 3c), which may be caused by low enrichment or a discrepant junction motif of F-circEA-2a. These results provided a rebuttal that F-circEA-4a is a promising biomarker for *EML4-ALK*-v3b translocation, highlighting its uncontroversial role in the liquid biopsy of *EML4-ALK*-positive NSCLC patients.

In summary, a novel circRNA F-circEA-2a produced from the *EML4-ALK* fusion gene was identified and mainly located in the cytoplasm to promote cell migration and invasion in lung cancer cells.

## Additional files

Additional file 1: **Table S1.** Information of primers and oligonucleotides used in this study. (DOCX 16 kb)

Additional file 2: Experimental materials and methods in this study. (DOCX 92 kb)

## Abbreviations

circRNAs: Circular RNAs;
EML4-ALK: Echinoderm Microtubule-associated protein-Like 4-Anaplastic Lymphoma Kinase;
NSCLC: Non-small cell lung cancer
qPCR: Real-time quantitative polymerase chain reaction
RT-PCR: Reverse transcription polymerase chain reaction

## References

[1] Siegel RL, Miller KD, Jemal A (2016). Cancer statistics, 2016. CA Cancer J Clin.

[2] Gridelli C, Rossi A, Carbone DP, Guarize J, Karachaliou N, Mok T (2015). Non-small-cell lung cancer. Nat Rev Dis Primers.

[3] Soda M, Choi YL, Enomoto M, Takada S, Yamashita Y, Ishikawa S (2007). Identification of the transforming EML4-ALK fusion gene in non-small-cell lung cancer. Nature.

[4] Mano H (2008). Non-solid oncogenes in solid tumors: EML4-ALK fusion genes in lung cancer. Cancer Sci.

[5] Horn L, Pao W (2009). EML4-ALK: honing in on a new target in non-small-cell lung cancer. J Clin Oncol.

[6] Rybak-Wolf A, Stottmeister C, Glazar P, Jens M, Pino N, Giusti S (2015). Circular RNAs in the mammalian brain are highly abundant, conserved, and dynamically expressed. Mol Cell.

[7] Guarnerio J, Bezzi M, Jeong JC, Paffenholz SV, Berry K, Naldini MM (2016). Oncogenic role of fusion-circRNAs derived from cancer-associated chromosomal translocations. Cell.

[8] Tan S, Gou Q, Pu W, Guo C, Yang Y, Wu K (2018). Circular RNA F-circEA produced from EML4-ALK fusion gene as a novel liquid biopsy biomarker for non-small cell lung cancer. Cell Res.

[9] Han D, Li J, Wang H, Su X, Hou J, Gu Y, Qian C (2017). Circular RNA MTO1 acts as the sponge of miR-9 to suppress hepatocellular carcinoma progression. Hepatology.

[10] Li Y, Zheng Q, Bao C, Li S, Guo W, Zhao J (2015). Circular RNA is enriched and stable in exosomes: a promising biomarker for cancer diagnosis. Cell Res.

