# Supplementary material for: Circular RNA F-circEA-2a derived from EML4-ALK fusion gene promotes cell migration and invasion in non-small cell lung cancer
Source: Mol Cancer. 2018 Sep 20;17:138. doi: 10.1186/s12943-018-0887-9 (PMC6146612; doi:10.1186/s12943-018-0887-9)
Supplement: Supplementary file 1 — Table S1. Information of primers and oligonucleotides used in this study. (DOCX 16 kb) [file 12943_2018_887_MOESM1_ESM.docx]

**Table S1, Information of primers and oligonucleotides used in this study:**

| **Primers for identification of F-circEA-4a and F-circEA-2a:**  F1: GCAGAGCCCTGAGTACAAGC  R1: GCTTGGTTGATGATGACATCTTTATG |
| --- |
| **Primers for identification of *EML4-ALK* fusion gene (AB374362.1):**  F2: TACCAGTGCTGTCTCAATTGCAG  R2:TGCCAGCAAAGCAGTAGTTG |
| **Nested primers for F-circEA-4a:**  F3: CAACTACTGCTTTGCTGGCA  R3: AGGTGATGCTCGAATTTTCAGAG |
| **Nested primers and qPCR primers for F-circEA-2a:**  F4: CAACTACTGCTTTGCTGGCA  R4: TGCTCGAATTCAGAGCACAC |
| **Primers for the establishment of F-circEA-2a-overexpressing plasmid:**  F5: ATTTTTTTTATTTTATGCAGATTCGAGCATCACCTTCTCCCCAGC  R5: TTGGAATTTTGAATACTTACTCAGAGCACACTTCAGGCAGCGTC  F6: CCTCCATAGAAGATTCTAGAAGACCCAAGCTGGCTAGCG  R6: CGCAGATCCTTCGCGGCCGCTCGAGGCTGATCAGCGGGT |
| **Primers for identification of *GAPDH* mRNA:**  F7: ACCACAGTCCATGCCATCAC  R7: TCCACCACCCTGTTGCTGTA |
| **Oligo-nucleotide junction probe for F-circEA-4a in dot blotting hybridization:**  GTGATGCTCGAATTTTCAGAGCACACTTCAGG  **Oligo-nucleotide junction probe for F-circEA-2a in dot blotting hybridization:**  GTGATGCTCGAATTCAGAGCACACTTCAGGC |
| **F-circEA-2a sequence:**  ATTCGAGCATCACCTTCTCCCCAGCCCTCTTCACAACCTCTCCAAATACACAGACAAACTCCAGAAAGCAAGAATGCTACTCCCACCAAAAGCATAAAACGACCATCACCAGCTGAAAAGTCACATAATTCTTGGGAAAATTCAGATGATAGCCGTAATAAATTGTCGAAAATACCTTCAACACCCAAATTAATACCAAAAGTTACCAAAACTGCAGACAAGCATAAAGATGTCATCATCAACCAAGCAAAAATGTCAACTCGCGAAAAAAACAGCCAAGTGTACCGCCGGAAGCACCAGGAGCTGCAAGCCATGCAGATGGAGCTGCAGAGCCCTGAGTACAAGCTGAGCAAGCTCCGCACCTCGACCATCATGACCGACTACAACCCCAACTACTGCTTTGCTGGCAAGACCTCCTCCATCAGTGACCTGAAGGAGGTGCCGCGGAAAAACATCACCCTCATTCGGGGTCTGGGCCATGGCGCCTTTGGGGAGGTGTATGAAGGCCAGGTGTCCGGAATGCCCAACGACCCAAGCCCCCTGCAAGTGGCTGTGAAGACGCTGCCTGAAGTGTGCTCTGA |
