# Supplementary material for: Circular RNA F-circEA-2a derived from EML4-ALK fusion gene promotes cell migration and invasion in non-small cell lung cancer
Source: Mol Cancer. 2018 Sep 20;17:138. doi: 10.1186/s12943-018-0887-9 (PMC6146612; doi:10.1186/s12943-018-0887-9)
Supplement: Supplementary file 2 — Experimental materials and methods in this study. (DOCX 92 kb) [file 12943_2018_887_MOESM2_ESM.docx]

**Experimental Materials and Methods in this study**

**Plasmid construction**

To construct the F-circEA-2a-overexpressing plasmid, the sequence including partial exon 4-6 of EML4 gene and the partial exon 20-22 of ALK gene was amplified from cDNAs of H2228 cells, and inserted into the vector pcDNA3.1-Laccase2 MCS Exon at *Age*I site by One Step Cloning Kit (Vazyme), then both the cloned F-circEA-2a sequence and Laccase2 insert sequence were cloned into pCDH-CMV- MCS-EF1-puro (System Biosciences) at *Xba*I*/Not*I sites. Primer sequences were listed in the Table S1.

**Blood sample collection and RNA preparation**

The lung tissues and blood samples from NSCLC patients were collected at West China Hospital, which were approved by the Ethics Committee of West China Hospital of Sichuan University, China. Written informed consent for research purposes was provided for the patients. The plasma was separated at 1,500 *rpm* for 10 min at room temperature within two hours after blood collection in EDTA-coated Vacutainer tubes. The separated plasma was subjected to total RNA isolation by TRIzol^TM^ LS reagent (Invitrogen).

**Cell culture and transfection**

H2228 cells were purchased from Cobioer Biological Corporation (NanJing, China). H2228, H1299 and A549 cells were cultured in RPMI-1640 medium (Gibco) plus 10% fetal bovine serum (FBS) and 1% penicillin/streptomycin in an incubator with 5% CO_2_. Cells were transfected with Lipofectamine® 2000 Reagent (Invitrogen) according to the manufacture’s instructions.

**Cellular nucleus/cytoplasm fractionation**

Cells were harvested and washed twice with phosphate buffer saline (PBS), then resuspended in the RLN buffer (50 mM Tris-HCl, pH 7.0, 140 mM NaCl, 1.5 mM MgCl_2_, 0.5% NP-40), and incubated on ice for 1 min. Cell lysates were centrifuged at 300 g for 3 min at 4°C to collect the supernatants as the cytoplasmic fraction. The remaining pellets were washed twice with RLN buffer, and then resuspended in RLN buffer as the nucleic fraction.

**RNA isolation, F-circEA-2a identification and quantitative RT-PCR**

Total RNAs were extracted from cells or patient tissues by TRIzol^TM^ reagent (Invitrogen) according to the manufacture’s instructions. For circular RNA identification, total RNAs (5 μg) were incubated with 15 units of Ribonuclease R (RNase R, Epicentre Technologies) at 37°C for 15 min to digest linear RNAs, then precipitated by ammonium acetate and glycogen, followed by phenol/chloroform extraction. The RNAs were subjected to reverse transcription by random primers using the RETROscript**^®^** kit (Life Technologies). Then PCR reactions were performed using Phanta**^®^** Max Super-Fidelity DNA Polymerase (Vazyme) and divergent primers, which were listed in Table S1. F-circEA-2a and F-circEA-4a were detected from patients’ samples using nested PCR (first round PCR primers: F1/R1; nested PCR primers for F-circEA-4a: F3/R3, nested PCR primers for F-circEA-2a: F4/R4). For quantitative real-time PCR, cDNAs were generated by M-MLV Reverse Transcriptase Kit (Life Technologies), and qPCR was performed with SYBR Green Master Mix using StepOne Plus real-time PCR system (Applied Biosystems). Primer sequences were listed in the Table S1. To evaluate the enrichment of F-circEA-2a, GAPDH mRNA, U6 RNA in nuclear and cytoplasmic fractions, subtract the C_T_ value in the cytoplasmic fraction from the C_T_ value in the nuclear fraction to obtain the ΔC_T_ value. Then use the following formula to calculate the relative ratio of RNAs in nuclear and cytoplasmic fractions:


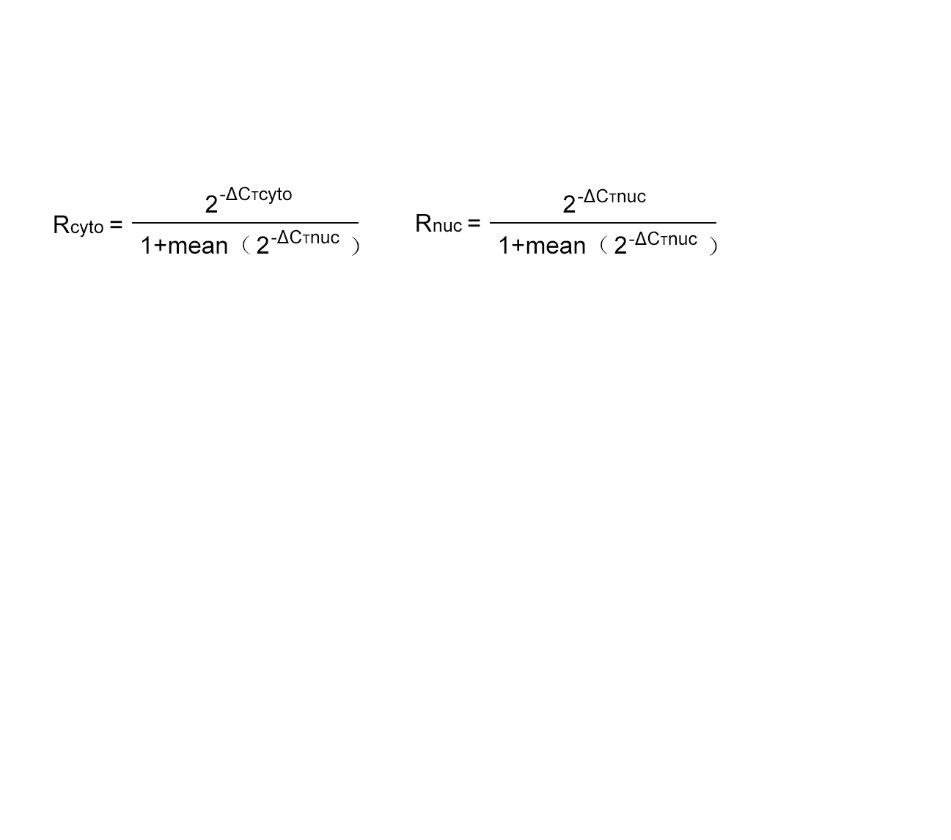


**Dot blot hybridization**

Oligo-nucleotide probes were end-labeled by T4 polynucleotide kinase (NEB) with [γ-^32^P]-ATP (PerkinElmer). RNA samples were subjected onto nitrocellulose membranes, cross-linked at 120,000 mJ/cm^2^ with Stratalinker UV Crosslinker (Stratagene). Pre-hybridization was conducted in the hybridization buffer (Life Technologies) at 42°C for 1 h, followed by hybridization overnight at 42°C with γ-^32^P labeled probes. The membranes were subsequently washed once twice with 1 x SSC plus 0.1% SDS for 15min at 50°C. Finally, the hybridization signals were detected by Typhoon FLA 7000 Phosphor Imager. The oligo-nucleotide probes were listed in the Table S1.

**Transwell migration and invasion assay**

Cell migration assays were performed using Transwell chamber (Millipore), and cell invasion assays were done with chambers uniformly covered with Matrigel (BD Biosciences) diluted with RPMI-1640 medium (1:7). Cells were suspended in RPMI-1640 medium containing 5% BSA and seeded into the top chamber, while RPMI-1640 medium supplemented with 10% FBS and 1% penicillin/streptomycin were added into the bottom chamber as chemoattractant. After incubation at 37°C for 24 hours, migration was terminated by swabbing cells in the top chamber with a cotton swab. Cells present at the lower surface of the membrane were fixed with 4% paraformaldehyde for 20 min, stained with 1% crystal violet (Sigma) for 15 min. The cells were counted in at least six randomly selected microscopic fields under an inverted phase-contrast microscope. The experiment was repeated three time independently.

**Wound healing assay**

Cells were cultured in 6-well plate and wounded by a sterilized pipet tip to make a straight scratch. After being rinsed with PBS gently, cells were incubated in RPMI-1640 medium plus 0.5% FBS and 1% penicillin/streptomycin. Pictures were taken by an Olympus digital camera for every 6 hours.

**MTT and colony formation assays**

Cells were seeded into 96-well plate for MTT assay. At the indicated time points, the cells were incubated with 0.5 mg/ml sterile MTT (Beyotime, Shanghai, China) at 37℃ for 4 hours, then the media were removed and replaced with 150 μL DMSO. The absorbance was measured at 570 nm. For colony formation assay, cells were seeded into 6-well plate and cultured for 5 days, then the cells were fixed with 4% paraformaldehyde for 20 min, stained with 1% crystal violet (Sigma) for 20 minutes. After removing crystal violet solution, the cells were washed with water and colony formation was recorded by high-resolution scanner. All experiments were performed in triplicates.
